# Towards a structural biology work bench

**DOI:** 10.1107/S090744491300276X

**Published:** 2013-04-19

**Authors:** Chris Morris

**Affiliations:** a STFC Daresbury Laboratory, Warrington, England

**Keywords:** software, integrative structural biology, structural biology

## Abstract

This is an introduction to four papers based on presentations given at a workshop entitled Integrated Software for Integrative Structural Biology. The use of hybrid techniques, and other trends in structural research, pose new challenges to software developers. A structural biology work bench that meets these needs would provide seamless data transfer between processing steps, and accumulate archival data and metadata without intruding into the scientist’s work process.

## Abstract 2

The trends to investigate larger complexes and more transient phenomena pose some challenges to software developers.

Four papers in this issue of *Acta Crystallographica Section D* are based on presentations given at a workshop entitled Integrated Software for Integrative Structural Biology organ­ised by the Computational Centre for Integrative Structural Biology (CCISB) on 21–23 May 2012.

This workshop discussed some new challenges which structural biologists are accepting. They are addressing larger macromolecular machines rather than single gene products (Perrakis *et al.*, 2011 ▶); investigating movement of molecules rather than snapshots; and processing data that is heterogeneous, noisy and incomplete. Karaca & Bonvin (2013 ▶) say ‘understanding how a single cell functions is the fundamental quest of life sciences’. This can only be comprehensively addressed once the structure–function relationships of biomolecular complexes occurring in that particular cell will have been explored thoroughly. There are two main classical experimental techniques that can reveal the structure of the biomolecular complexes in atomistic detail: X-ray crystallography and NMR spectroscopy. Although these have helped immensely to shed light on the mechanical and functional world of biomolecules, they are faced with many challenges when the biomolecular systems under study become very large, comprise flexible or unstructured regions, exist in very tiny amounts, are membrane associated, or when their constituents interact only transiently.

Most structural biologists are expert in one or two tech­niques. Many now spend part of the year working as a novice, using a technique which they have not yet mastered to provide supplementary evidence about their target. Indeed, the Instruct visit mechanism is designed to encourage such work. The paper by Karaca & Bonvin ▶ provides an example of software support for such hybrid work, as they ‘have integrated low-resolution shape data obtained from either ion mobility mass spectrometry (IM-MS) or SAXS experiments, into the conventional scoring function of our information-driven docking program *HADDOCK*’. The software described by Marabini *et al.* (2013 ▶) offers, among other things, ‘the visualization of maps obtained from 3D-EM, together with annotations provided by other techniques’.

Biasini *et al.* (2013 ▶) explain that ‘research projects in structural biology increasingly rely on combinations of heterogeneous sources of information, *e.g.* evolutionary information from multiple sequence alignments, experimental evidence in the form of density maps, or proximity constraints from proteomics experiments … new methods in computational structural biology often rely on custom-made *ad hoc* combinations of command-line tools built to perform specific tasks’. They present current developments in the computational structural biology framework *OpenStructure*, which supports the integration of information from a variety of origins.

Structural biologists want to deliver their results to other life scientists, including systems biologists and medicinal chemists. Gutumanas *et al.* (2013 ▶) explain that ‘it seems unavoidable that the role of structural biology archives will change from being a pure repository of historic data into becoming an indispensable resource for the wider biomedical community. As part of this transformation, it will be necessary to validate the biomacromolecular structure data and ensure the highest possible quality for the archive holdings; to combine structural data from different spatial scales into a unified resource; and to integrate structural data with functional, genetic and taxonomy data as well as other information available in bioinformatics resources’.

Arising from these scientific challenges, there are several challenges to the software developers whose work supports structural biology. One is to improve the collection of data and metadata for archiving. Another is to improve the automatic processing pipelines used in protein crystallography, and to create pipelines in disciplines that do not yet have them, to support the work of ‘visiting scientists’ from other subdisciplines. Complementary to this, in cases where automated processing is insufficient, it is necessary to better record the information that scientists provide, to make the processing truly reproducible.

Even more challenging is the development of algorithms that can combine data from different techniques, including improved integration of experimental methods with modelling; and to provide some indication of the reliability of the results, represented by an effective visualization.

A structural biology work bench that meets these needs would provide seamless data transfer between processing steps, and accumulate archival data and metadata without intruding into the scientist’s work process.

For a crystallographic group, the effort required to install the *CCP*4 suite is repaid many times. If one of them needs to process a few SAXS results, the effort of installation will be a more significant overhead. The work bench must support a very broad range of techniques, and so it should be available without any installation at all: all its functionality should be available through a website.

The internal organization of the work bench must be open to the addition of new algorithms, without imposing particular architectural choices on the algorithm developers. The architectural design should also take good note of the point that Marabini *et al.* (2013 ▶) make that ‘developers can never quite know what requirements will be needed to accommodate the different algorithms of each software package, so high flexibility is mandatory. As the data become more complex and heterogeneous, an ontological database is probably the best option’.

The scientific community includes a broad group of life scientists who use structural results without much attention to the underlying physics, and a small number of pioneers who develop new structural algorithms. There is also a significant intermediate group: there are many structural biologists who are highly competent in computing and who write scripts to plumb together the executables they use in novel ways. The work bench must serve all three groups.

Facebook supports its users in developing and sharing new applications (or apps). ‘Google widgets’ provide similar functionality. The work bench should similarly make it easy to develop and share new functional web pages, to provide novel structural and bioinformatic functionality. Important steps have already been taken in this direction, for example by weNMR, *Scipion*, and the archiving performed at Diamond Light Source. Any future collaboration must build on existing achievements, not duplicate them.

Developing infrastructure software that supports the daily work of a broad community needs a different approach to developing new algorithms. Experience shows that success depends on an effort to understand the context of use and then more detailed requirements, to take into account the user experience, and then design a software architecture (Morris & Segal, 2012 ▶). This will take a significant effort, which will provide structural biologists with the software tools they need to continue their extraordinary progress in rate of discovery.
